# Aumolertinib plus bevacizumab for untreated advanced NSCLC with EGFR sensitive mutation

**DOI:** 10.3389/fonc.2025.1595812

**Published:** 2025-06-04

**Authors:** Lingping Kong, Lina Peng, Xue Yang, Qing Ma, Linlin Zhang, Xia Liu, Diansheng Zhong, Fanlu Meng

**Affiliations:** Department of Medical Oncology, Tianjin Medical University General Hospital, Tianjin, China

**Keywords:** aumolertinib, bevacizumab, EGFR-TKI, non-small cell lung cancer, progression-free survival

## Abstract

**Background:**

Aumolertinib is a novel third-generation epidermal growth factor receptor tyrosine kinase inhibitor (EGFR-TKI) with proven efficacy and safety for untreated non-small-cell lung cancer (NSCLC) patients with EGFR sensitizing mutations (EGFRm) in China. The progression-free survival (PFS) improvement of the combination of first-generation EGFR-TKIs and bevacizumab was confirmed by CTONG1509, JO25567, and NEJ026 studies, however, the effect of third-generation EGFR-TKIs plus bevacizumab remains under debate. This study aimed to investigate the efficacy and safety of aumolertinib plus bevacizumab in untreated EGFRm advanced NSCLC.

**Methods:**

We conducted a phase II single-arm prospective clinical trial for advanced EGFRm NSCLC treated with aumolertinib combined with bevacizumab. Treatment continued until disease progression, occurrence of unacceptable toxicities, or the patient withdrew consent. The study was stratified according to sex, smoking history, stage, EGFR mutation status, and central nervous system (CNS) metastasis. The primary endpoint was the 12–month progression-free survival rate (PFS%), and secondary endpoints included the objective response rate (ORR), overall survival (OS), and progression-free survival (PFS).

**Results:**

Between September 16, 2020, and November 11, 2021, a total of 21 patients were enrolled in the study. The median follow-up was 36.8 months (ranging from 33.2 to 40.4 months), and all 21 patients were included in the evaluation. The PFS% at 12-month was 81% (95% confidence interval (CI): 64.1–97.9%), the median PFS was 26 months (95% CI: 16.5-35.5) and the ORR reached 85.7%, with an average reduction of the target lesions of 48.2%. Among patients with CNS metastasis, the ORR was 92.9% (13/14), and for TP53 co-mutation patients, the ORR was 86.6% (12/14). Grade 3 adverse events were observed in 4 patients (19.2%), and no grade 4 or 5 adverse events reported.

**Conclusion:**

The combination of aumolertinib and bevacizumab in patients with advanced EGFRm NSCLC achieved the study’s primary endpoint. This study indeed extended PFS compared with previous literature, and it was deemed safe and tolerable.

## Introduction

Approximately 20-40% of non-small-cell lung cancer (NSCLC) patients harbor epidermal growth factor receptor mutations (EGFRm), primarily consisting of exon 19 deletions and exon 21 p.L858R (L858R) mutations (1). Epidermal growth factor receptor tyrosine kinase inhibitors (EGFR-TKIs), including gefitinib, erlotinib, and osimertinib, are the preferred first-line treatment for patients with EGFR mutations (2–4). Notably, when employed as the initial treatment for EGFRm NSCLC patients, osimertinib has demonstrated prolonged progression-free survival (PFS) and overall survival (OS) compared to erlotinib and gefitinib (2, 5). Furthermore, studies have indicated its superior brain permeability compared to first- and second-generation treatments (6, 7). Nevertheless, despite these advancements, disease progression and acquired resistance remain inevitable.

To address this challenge, integrated treatment strategies have been proposed. Bevacizumab, a recombinant anti-vascular endothelial growth factor (VEGF) monoclonal antibody, functions by inhibiting angiogenesis and impeding tumor growth (8). In numerous clinical phase 2/3 trials, the combination of EGFR-TKIs and bevacizumab has consistently shown a significant enhancement in progression-free survival (PFS) compared to EGFR-TKIs monotherapy when used as the primary treatment for patients with EGFRm NSCLC (9–11). Various phase 3 studies, such as FLAURA2 and AENEAS, which compare third-generation TKIs with first-generation TKIs as the initial therapy for patients with advanced EGFRm NSCLC, have reported the superior PFS associated with third-generation agents. Consequently, the exploration of combining third-generation EGFR-TKIs with bevacizumab in the first-line treatment of EGFRm NSCLC represents a current focal point of research.

Aumolertinib (formerly known as almonertinib; HS-10296) is an oral, third-generation EGFR-TKI designed to selectively target mutant EGFR rather than wild-type EGFR. It has been specifically formulated for the treatment of advanced EGFRm NSCLC. The APPOLO study demonstrated the remarkable efficacy and safety profile of aumolertinib in patients with EGFR T790M-positive NSCLC, showcasing its effectiveness, particularly in brain metastases (BM) (13). In the AENEAS trial, a phase 3 study conducted among Chinese patients, aumolertinib, as a first-line treatment, significantly extended progression-free survival (PFS) and duration of response (DOR) when compared to gefitinib in patients with advanced EGFRm NSCLC. However, the overall survival (OS) data from this study are currently immature (12).

A phase 1/2 single-arm study evaluating the initial treatment of osimertinib plus bevacizumab for advanced EGFRm NSCLC patients successfully achieved its primary endpoint, which was the 12-month progression-free survival rate (PFS%) (14). Building upon this foundation, we propose an investigation into the combination of aumolertinib and bevacizumab as a first-line therapy for advanced EGFRm NSCLC. Our objective is to explore the efficacy and safety of aumolertinib plus bevacizumab in untreated EGFRm advanced NSCLC.

## Methods

### Patient selection

Patients were enrolled between September 2020 to November 2021, and followed up until August 2024. The eligibility criteria were as follows: (1) individuals aged 18 years or older, (2) NSCLC confirmed histologically/cytologically or diagnosed clinically as advanced peripheral lung cancer, (3) EGFR-sensitizing mutation identified through next-generation sequencing (NGS) analysis, (4) clinical stage IIIB, IIIC, or IV disease not suitable for radical radiation therapy (based on the eighth edition of lung cancer TNM staging system), (5) presence of measurable lesion according to the Response Evaluation Criteria in Solid Tumors (RECIST) version 1.1; (6) adequate hematologic and organ function; (7) Eastern Coop-erative Oncology Group performance status 0 or 1, (8) signed informed consent form.

The exclusion criteria were: (1) previous exposure to EGFR inhibitors or VEGF receptor inhibitors, (2) coexistence or history of interstitial lung disease, (3) a high susceptibility to bleeding or embolism, (4) unmanaged hypertension, (5) Patients who received radiotherapy to the brain can participate, but are required to have an interval ≥14 days between the last days of radiotherapy and study treatment.

### Treatment

The patients received oral aumolertinib (110 mg) once daily and bevacizumab (Avastin, 7.5 mg/kg) on day 1 intravenously every 3 weeks. Treatment persisted until disease progression, occurrence of unacceptable toxicities, or withdrawal of patient consent.

### Evaluation of efficacy and safety

Eight weeks following the initiation of aumolertinib and bevacizumab, routine chest/abdominal computed tomography and brain magnetic resonance imaging (MRI) were conducted, with subsequent assessments every 2-4 months. Tumor lesion response was evaluated based on the Response Evaluation Criteria in Solid Tumors (RECIST) version 1.1. The Objective Response Rate (ORR) was defined as the proportion of patients achieving complete response and partial response (CR+PR). The Disease Control Rate (DCR) was calculated as the proportion of patients demonstrating objective response or stable disease for a minimum of four weeks. PFS was delineated as the duration from the initiation of the first medication to disease progression or death. The primary endpoint focused on 12-month PFS%, while secondary endpoints encompassed ORR, PFS, and safety parameters.

Adverse events (AEs) were systematically evaluated throughout the study, graded in accordance with the National Cancer Institute Common Terminology Criteria for Adverse Events version 5.0.

### Sample size calculation

To consider combined aumolertinib and bevacizumab a promising treatment strategy, we aimed for an improvement in 12-month PFS rates from a historical 60% to 80%, which would require a sample size of 24 patients to provide 80% power while controlling the type 1 error at 10%. Ultimately, we achieved 80% of our projected enrollments.

### Statistical analysis

Survival analyses were performed according to the Kaplan-Meier method, and confidence intervals (CIs) were calculated at a 95% confidence level. All analyses were performed using IBM SPSS software, version 24.0.

### Ethics

This study adhered to the rules and regulations of clinical studies with respect to human subject protection, and it was approved by the Tianjin Medical University of General Hospital of Ethics Committee and conducted in accordance with the principles of the Declaration of Helsinki. Informed consent was obtained from all enrolled patients.

## Results

### Patient characteristics

A total of 21 patients, comprising four men and fifteen women, were included in this study conducted between September 16, 2020, and November 11, 2021. The detailed characteristics of these enrolled patients are presented in **Table 1**. The median age of the participants was 62 years, with an age range spanning from 41 to 89 years. The majority of patients presented with adenocarcinoma (85.7%), while one had squamous cell carcinoma, another had NSCLC (undetermined pathological subtype), and one patient clinically manifested advanced peripheral lung cancer. All patients were diagnosed at clinical stage IV and received primary treatment. The EGFR mutations at diagnosis were located in exons 19 deletion (42.9%) and 21 L858R (57.1%). TP53 mutation and primary T790M occurred in 71.4% and 19.0% of cases, respectively. 14 patients had concomitant CNS involvement, with 21.4% (3/14) presenting symptomatic brain metastases at enrollment. Brain radiotherapy was administered to two patients, one undergoing whole-brain radiotherapy (WBRT), and the other receiving gamma knife radiosurgery.

**Table 1:** Patient characteristics.

Characteristics	Variables	N
Total case		21
Age	Median(range), y	62 (41-89)
Sex	Male	4 (19%)
	Female	17 (81%)
Smoking	Yes	5 (23.8%)
	No	16 (76.2%)
Histology	Adenocarcinoma	18 (85.7%)
	Squamous cell carcinoma	1 (4.8%)
	NSCLC, unspecified type	1 (4.8%)
	Peripheral lung cancer	1 (4.8%)
Clinical stage	IV	21 (100.0%)
EGFR mutation	Exon 19 del	9 (42.9%)
	Exon 20 L858R	12 (57.1%)
T790M mutation	Yes	4 (19.0%)
	No	17 (81.0%)
TP53 mutation	Yes	15 (71.4%)
	No	6 (28.6%)
CNS	Yes	14 (66.7%)
	No	7 (33.3%)
Neurological symptoms	Yes	3 (21.4%)
	No	11 (78.6%)
Brain radiotherapy	Yes	2 (14.3%)
	No	12 (85.7%)
CEA	Abnormal	18 (85.7%)
	Normal	3 (14.3%)

CNS, central nervous system; EGFR, epidermal growth factor receptor; CEA, carcino-embryonic antigen.

### Clinical responses

**Figure 1:**
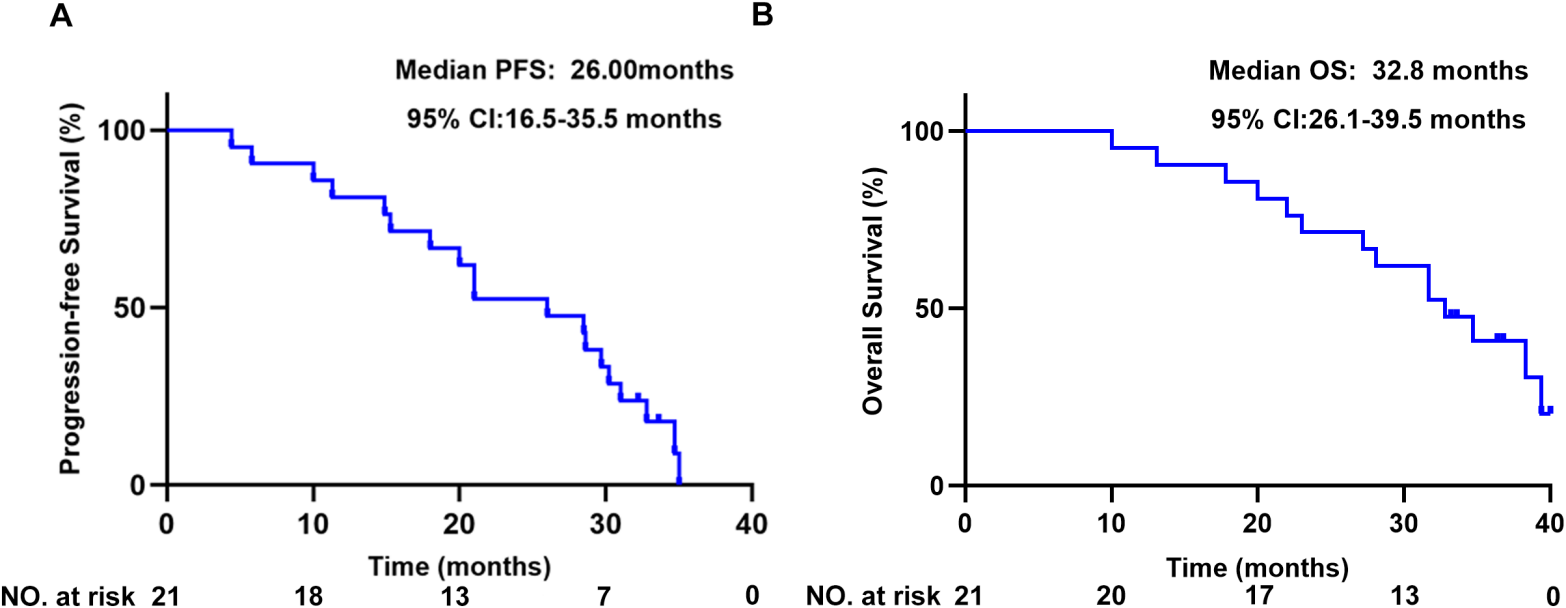
Kaplan-Meier estimates of **(A)** PFS, and **(B)** OS.

All 21 patients were ultimately evaluated. The primary endpoint, the 12-month PFS%, reached 81% (95% CI: 64.1–97.9%). The patients exhibited a median PFS of 26 months (95% CI: 16.5-35.5) (**Figure 1A**), with a median follow-up for PFS of 36.8 months (33.2m- 40.4m). The median OS was 32.8 months (95% CI: 26.1-39.5) (**Figure 1B**).

**Figure 2:**
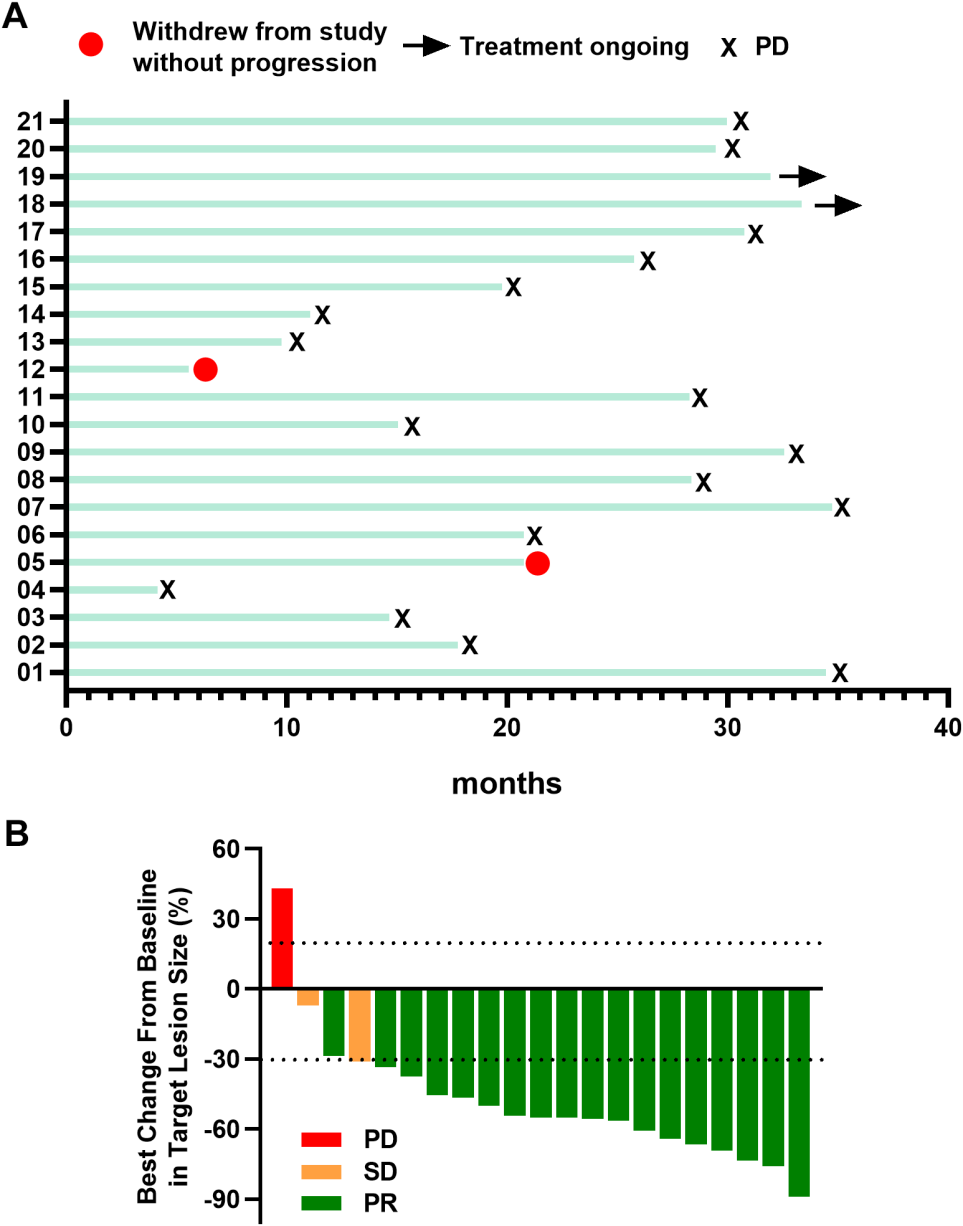
**(A)** Swimming plot of patients. **(B)** Best percentage change from baseline in target lesion size Responders were confirmed by Response Evaluation Criteria in Solid Tumors guidelines. PR, partial response; SD, stable disease; PD, progressive disease.

Within this cohort, seventeen patients experienced disease progression, whereas two withdrew from treatment without progression. Two patients continue to receive ongoing treatment (**Figure 2A**). Notably, 95.2% (20/21) of patients demonstrated a clinical response, marked by an average reduction of 48.2% in target lesions (**Figure 2B**).

**Figure 3:**
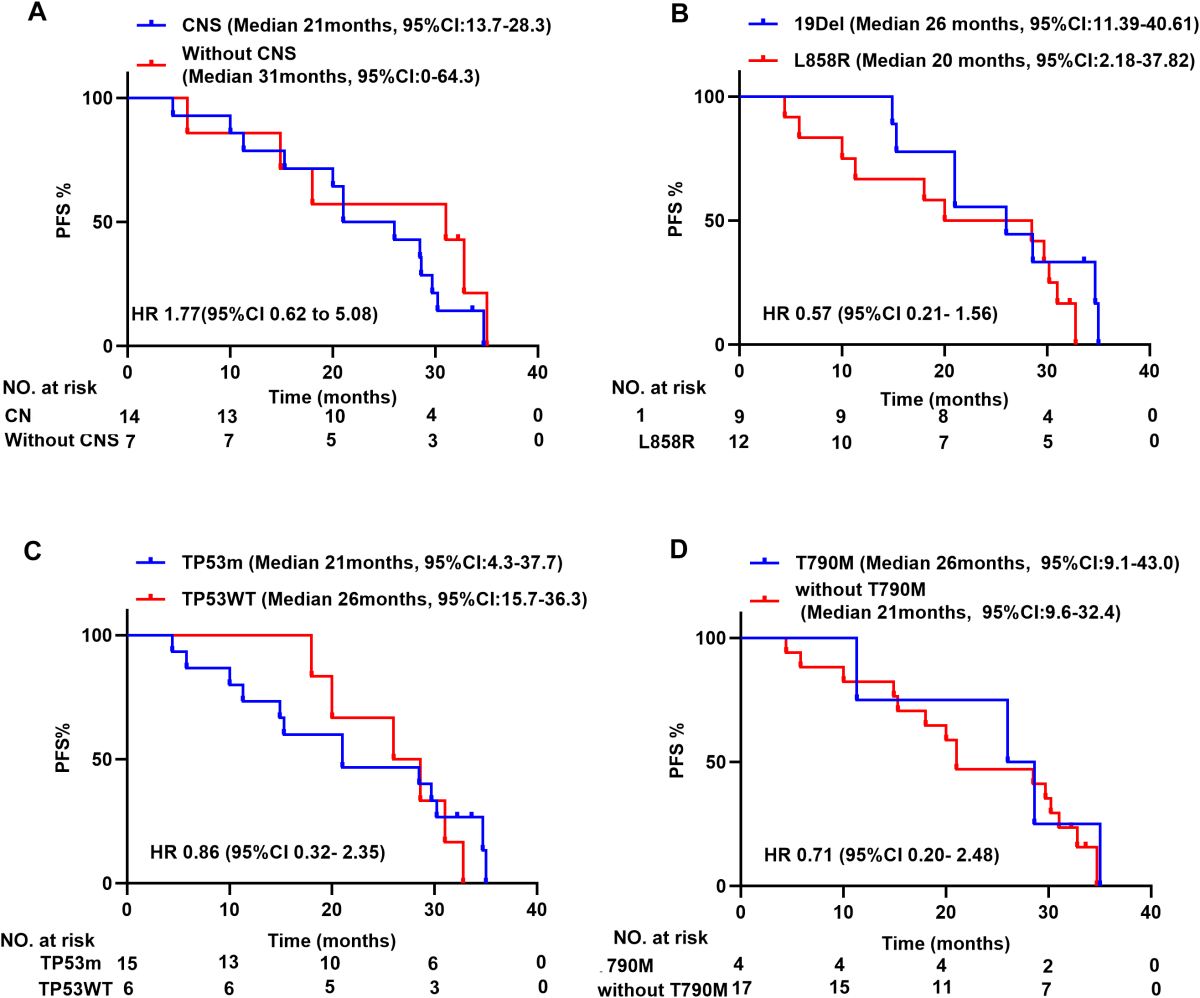
Subgroup analysis of PFS. **(A)**: 19Del and L858R. **(B)**: With or without CNS. **(C)**: with or without TP53 mutant. **(D)**: with or without T790M mutant. PFS, progression-free survival; 19 Del, EGFR 19 Del; L858R, EGFR 21 Exon L858R; CNS, central nervous system; TP53m, with TP53 mutant; TP53WT, without TP53 mutant; NE, not estimable; CI, confidence interval; HR, hazard ratio.

Subgroup analysis further explored various parameters. The median PFS for patients with CNS metastases (n=14) or without (n=7) were 21 (95% CI: 13.7-28.3) and 31 months (95% CI: 0–64.4), respectively, with a Hazard Ratio (HR) of 1.77(95% CI: 0.62–5.08) (**Figure 3A**). For patients with exon 19 deletion (n=9) or exon 21 p.L858 (n=12), the median PFS was 26 (95% CI: 11.4-40.6) and 20 months (95% CI: 2.2-37.8), respectively, with an HR of 0.57 (95% CI: 0.21–1.56) (**Figure 3B**). In patients with (n=15) and without (n=6) TP53 mutation, the median PFS was 21 months (95% CI: 4.34–37.67) and 26 months (95% CI: 15.68–36.32), respectively, with an HR of 0.86 (95% CI: 0.32–2.35) (**Figure 3C**). For patients with (n=4) and without (n=17) T790M mutation, the median PFS was 26 months (95% CI: 9.05–42.95) and 21 months (95% CI: 9.57–32.43), respectively, with an HR of 0.71 (95% CI: 0.20–2.48) (**Figure 3D**).

The ORR was 85.7% (18/21) (**Table 2**). Noteworthy ORRs were observed in patients with CNS metastasis (92.9%), intracranial lesions (90%), EGFR 19del (100.0%), EGFR L858R (75%), TP53 comutation (86.6%), and primary T790M comutation (100.0%) (**Table 2**).

**Table 2:** The response to aumolertinib and bevacizumab in subgroup analysis.

Response	Total N=21	CNS1 N=14	CNS2* N=10	19Del N=9	L858R N=12	TP53 N=15	T790M N=4
PR	18	13	9	9	9	13	4
SD	2	0	1	0	2	1	0
PD	1	1	0	0	1	1	0
ORR	18/21 (85.7%)	13/14 (92.9%)	9/10 (90%)	9/9 (100.0%)	9/12 (75%)	12/14 (86.6%)	3/3 (100.0%)
DCR	20/21 (95.2%)	13/14 (92.9%)	10/10 (100%)	9/9 (100.0%)	11/12 (91.7%)	14/15 (93.3%)	3/3 (100.0%)

* Eight patients have measureable intracranial lesions.

PR, partial response; SD, stable disease; PD, progressed disease; ORR, objective response rate; DCR, disease control rate; CNS, central nervous system.

### Safety

The AEs encountered by patients during treatment with aumolertinib and bevacizumab are delineated in **Table 3**. The predominant AEs included increased creatine phosphokinase (28.6%), proteinuria (19.0%), elevated AST/ALT levels (19.0%), and weakness (14.3%). AEs of grade 3 or higher severity were noted in four patients (19%), with no occurrences of grade 4 or 5 events. Bevacizumab discontinuation was necessitated in four patients (23.5%) due to bleeding risk or creatine phosphokinase elevation accompanied by chest pain and arrhythmia.

**Table 3:** Adverse events (AEs) graded according to the common toxicity criteria for adverse events (CTCAE).

AEs	All	Grade 1-2 (%)	Grade 3 (%)	Grade 4 (%)
Hematological				
Leucopenia	1 (4.8)	1 (4.8)	0 (0)	0 (0)
Nonhematological				
Rash	5 (23.8)	5 (23.8)	0 (0)	0 (0)
Pruritus	1 (4.8)	1 (4.8)	0 (0)	0 (0)
Anorexia	1 (4.8)	1 (4.8)	0 (0)	0 (0)
Constipation	1 (4.8)	1 (4.8)	0 (0)	0 (0)
Fatigue	3 (14.3)	3 (14.3)	0 (0)	0 (0)
Proteinuria	4 (19.0)	4 (19.0)	0 (0)	0 (0)
Oral mucositis	1 (4.8)	1 (4.8)	0 (0)	0 (0)
Toothache	1 (4.8)	1 (4.8)	0 (0)	0 (0)
Oral bleeding	2 (9.5)	2 (9.5)	0 (0)	0 (0)
Epistaxis	2 (9.5)	2 (9.5)	0 (0)	0 (0)
Hypertension	2 (9.5)	2 (9.5)	0 (0)	0 (0)
Chest pain	1 (4.8)	0 (0)	1 (4.8)	0 (0)
Myalgia	1 (4.8)	0 (0)	1 (4.8)	0 (0)
ALT/AST elevation	4 (19.0)	4 (19.0)	0 (0)	0 (0)
CPK elevation	6 (28.6)	4 (19.0)	2 (9.6)	0 (0)

ALT, alanine aminotransferase; AST, aspartate aminotransferase; CPK, Creatinine phosphokinase.

## Discussion

This phase 2 trial investigated the combined administration of aumolertinib and bevacizumab in previously untreated stage IV EGFRm NSCLC patients. This prospective Phase II clinical trial met the primary study endpoint. Moreover, in comparison with previously published literature, it demonstrated favorable treatment efficacy and exhibited good safety and tolerability. The adverse events align with the known characteristics of each agent.

The study design is rooted in prior trials showing improved efficacy and safety when combining first-generation EGFR-TKIs with VEGF inhibitors for EGFRm NSCLC patients (9–11, 16, 17). Previous trials revealed the significant improvement in PFS with third-generation TKIs as first-line treatment (2) (**Table 4**). Our study, notably, emulated the structure of a phase 1/2 trial investigating osimertinib and bevacizumab. The results showcased a 12-month PFS of 81%, surpassing the outcomes observed with aumolertinib alone and the osimertinib-bevacizumab combination. Compared with aumolertinib alone in AENEAS (19.3 months), the median PFS (26 months) in this study seemed to be more beneficial (12). During the period of our study, data from phase 3 studies of third-generation EGFR-TKIs and studies investigating osimertinib in combination with bevacizumab have been published and are summarized in **Table 4** (12, 14, 18–20). The WJOG9717L study, however, indicated no improvement in PFS with osimertinib-bevacizumab as first-line treatment (15). The mPFS and 1-year PFS rate in our study are higher than those in these studies, heralding the potential for the combination treatment regimen in this study to become a frontline therapeutic approach. It may require more refined stratification to identify specific populations that may precisely benefit from the combination therapy.

**Table 4:** Literature review of third-generation EGFR-TKIs for EGFRm NSCLC.

Trial	Treatment	N	CNS (%)	ORR (%)	PFS% (12mo)	mPFS (mo)
AENEAS (12)	Aumolertinib vs Gefitinib	214 215	26.2 27.4	73.8 72.1	66.2 37.9	19.3 9.9
FLAURA (2)	Osimertinib vs Gefitinib or Erlotinib	279 277	19.0 22.7	80 76	70 47	18.9 10.2
FULONG (18)	Furmonertinib vs Gefitinib	178 179	35 32	NA NA	NA NA	20.8 11.1
NCT03861156 (19)	Befotertinib vs Icotinib	182 180	32.4 31.7	75.8 78.3	NA NA	22.1 13.8
LASER301 (20)	Lazertinib vs Gefitinib	196 197	26 24	76 94	NA NA	20.6 9.7
NCT02803203 (14)	Osimertinib+ Bevacizumab	49	31	80	76	19
WJOG9717L (15)	Osimertinib± Bevacizumab	61 61	37.7 29.5	86 82	63.7 73.8	17.1 24.3

15, 21 were osimertinib plus bevacizumab as first-line treatment.

mPFS, median progression-free survival; PFS%, progress free survival rate; ORR, objective response rate; mo, months; N, patient numbers; CNS, central nervous system.

Our study’s patient characteristics differed, notably with a higher percentage of patients having preexisting CNS metastases (66.7%, **Table 4**) (2, 12, 18–20). CNS metastases associated with a more aggressive disease phenotype and significantly shorter TKI treatment-related PFS compared to those without CNS metastases (21). The established vascular normalization effect of antiangiogenic drugs in the central nervous system has therapeutic implications. The BRAIN study demonstrated that bevacizumab could delay brain metastases, showcasing its potential value (22). A retrospective study on EGFRm NSCLC patients with brain metastases revealed a 100% ORR and a 2-year survival rate of 62.5%, suggesting the efficacy of first-generation EGFR-TKI combined with bevacizumab as first-line therapy (23). Presently, third-generation EGFR-TKIs like osimertinib exhibit superior CNS efficacy in patients with EGFR-mutant brain metastases (24, 25). Our study aligns with the AENEAS analysis at ASCO 2022 (NO.9096), indicating that combining aumolertinib and bevacizumab may be effective and protective against CNS progression, as suggested by a 92.9% ORR in our study.

In subgroup analysis, patients with EGFR exon 19 deletions exhibited better PFS trends, contrasting with worse trends in those with EGFR L858R. This is consistent with the trend of subgroup analysis in the WJOG9717L study (15). The relationship between TP53 mutation and EGFR-TKI efficacy remains debated, our data indicate better PFS trends in patients without TP53 mutation. Additionally, previous studies indicated that the occurrence of a primary T790M mutation was associated with worse response and poor prognosis in patients with advanced EGFR-m NSCLC treated with first-generation (26–29), and second-generation (30, 31) EGFR-TKIs. In recent studies, primary T790M mutation showed some sensitivity towards osimertinib with the ORR fluctuating between 10% and 72.2% (32–37). In our study, four patients who had concurrent EGFR T790M at baseline benefited from the treatment strategy combining bevacizumab with aumolertinib. The best therapeutic outcome was PR, and the ORR reached 100%. This suggests that in cases of primary resistance to first-generation EGFR TKIs mediated by the primary EGFR T790M mutation, the treatment strategy adopted in this study is capable of enhancing the clinical efficacy.

Despite these therapeutic implications, our study has limitations. A small sample size from a single institution may impact result conclusiveness. Being a single-arm study lacks a control group, and future studies should expand the sample size and consider randomized controlled trials.

In conclusion, our study suggests potential benefits of aumolertinib and bevacizumab combination treatment for EGFRm advanced NSCLC compared to aumolertinib monotherapy. Nonetheless, it cannot be concluded arbitrarily that aumolertinib combined with bevacizumab is the first-line treatment of EGFRm NSCLC. Next, we should focus on subgroup analysis to select the population more suitable for the combination of aumolertinib and bevacizumab, further expand the sample size, and conduct randomized controlled studies. In the future, whether aumolertinib combined with bevacizumab can significantly improve PFS as a first-line treatment strategy may be answered.

## Data Availability

The raw data supporting the conclusions of this article will be made available by the authors, without undue reservation.
